# Characterization and Modeling Quality Analysis of Edible Oils Using Electrochemical Impedance Spectroscopy

**DOI:** 10.1155/2022/2781450

**Published:** 2022-08-21

**Authors:** T. A. Elmosalami, Mahmoud M. Kamel, I. Tomashchuk, Meshal Alzaid, Massaud Mostafa

**Affiliations:** ^1^Physics Department, College of Science, Jouf University, P. O. 2014, Sakaka, Saudi Arabia; ^2^Physics Department, Faculty of Science, Zagazig University, Zagazig, Egypt; ^3^Chemistry Department, College of Science, Jouf University, P.O. Box 2014, Sakaka 42421, Saudi Arabia; ^4^Laboratoire Interdisciplinaire Carnot de Bourgogne (ICB), UMR CNRS 6303-Université de Bourgogne-Franche Comté, 12, Rue de la Fonderie- 71200 Le Creusot, France; ^5^Physics Department, Faculty of Science, South Valley University, Qena 83523, Egypt

## Abstract

The dielectric characteristics of six culinary oils were measured over the frequency range of 0.01 Hz–100 kHz. The results showed that the dielectric constants of oils had the same frequency relationship (i.e., they decreased with increasing frequency). The dielectric constants at lower frequencies for olive oil A, olive oil B, sesame oil, Nigella sativa, sunflower oil, and corn oil are approximately 2.75, 2.5, 2.0, 1.75, 1.5, and 0.9. An FT-IR analysis showed that the spectral differences were very small, because most vegetable oils contain the same type of fatty acids. The model built using COMSOL Multiphysics for the potential and electric field distributions for different oils and used to calculate the dielectric constant was simulated under various conditions in the AC/DC module. The model results were compared with the experimental results, which showed satisfactory convergence between them. The experimental and model results obtained in this study could be useful for evaluating the edible oil quality.

## 1. Introduction

The organoleptic and nutritional benefits of extra virgin olive oils have been the driving force behind their global consumption in recent decades. The preservation and assurance of extra virgin olive oil quality throughout the commercial cycle are crucial for producers, merchants, and consumers [1]. During storage, the quality of extra virgin olive oil degrades due to oxidation, causing rancidity and hydrolytic degradation, resulting in partial loss of small components [2]. Olive oil companies use chemical and sensory tests to ensure the product quality. These tests verify that the olive oil product satisfies the criteria of the customers and the quality standards set by the International Olive Council (IOC). The impact of cooking oil on the health and fitness of humans can be easily identified. The oil quality impacts the skin and digestive system within a few days of use [3]. It directly affects the heart, blood sugar levels, and body fat percentage. This study proposes the creation of an interleaved capacitance sensor that can be used to determine fluctuations in oil capacitance by varying the frequency and temperature parameters. The dielectric constant of the oil sample was computed using the measured capacitance value [3].

Recent studies have described the use of various techniques to assess oil quality, including colorimetric reactions [4], total polar compound (TPC) measurement [5–8], and near-infrared spectroscopy [9]. The quality of fried food is associated with the oil and food used [10]. The water in frying foods reacts with air under the effect of temperature, causing chemical reactions such as oxidation, hydrolysis, and polymerization [11]. Peroxide is the principal result of oil chemical breakdown and contributes to nonvolatile polar molecules (PC) [12–14]. A capacitive sensor can be used to assess polar chemicals in oil [15, 16]. Oil permittivity is defined as the ratio of capacitance in oil to that in air and can be used as a quality indicator [17]. However, testing kits, such as Testo 270, Food Oil Monitor 310, and Capsens 5000, are expensive. Consequently, they are only utilized in high-end restaurants and the European food sector [18]. Sensory assessment techniques, column chromatography, chemical systems, and infrared detection methods are traditional methods for accurate quality analysis [19]. Several researchers have discussed these approaches over the years [20]. However, these methods are expensive, require expertise to operate the equipment, not portable, and time-consuming.

This study is aimed at investigating the effects of the frequency on the impedance and dielectric constant of various vegetable oils. In this study, a new simulation is developed to predict the electrical properties of various vegetable oils. Moreover, the relationship between experimentally defined dielectric properties and those from the simulation of olive and vegetable oils are discussed to improve it as a simple and useful method for oil quality monitoring during processing and storage.

## 2. Materials and Methods

**Figure 1:**
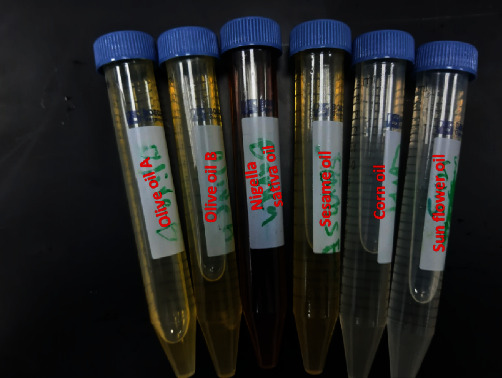
Oil samples used in the experiment.

The olive oils coded A and B were obtained from several locations in the Al Jouf region in the Kingdom of Saudi Arabia KSA. Both olive oils A and B were compared to Nigella sativa, sesame oil, corn oil, and sunflower oil, all of which were collected from marketplaces in the same area.  Figure 1 shows the different samples used in this study.  Table 1 lists their physicochemical properties, which were provided by the producers.

**Figure 2:**
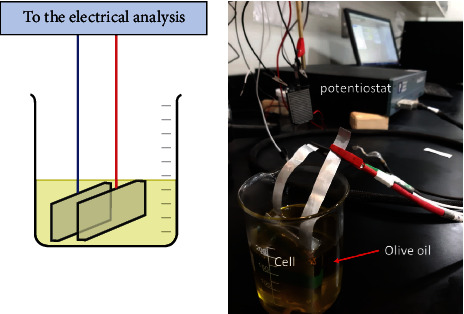
Illustration of the proposed olive oil dielectric constant measurement setup.

An AMETEK impedance analyzer (VersaSTAT4: model 400) was used to measure the dielectric parameters. The olive oil samples were placed in a beaker with an aluminum electrode with a diameter of 5 × 5 mm and a fluid gap of 1 mm in parallel mode, as shown in Figure 2. For all samples, the capacitance *C*_0_ of the empty cell, capacitance *C*_*aps*_ of the cell filled with oil, impedance *Z*, and capacitance *C*_*aps*_ were all measured in the frequency range of 0.001 Hz to 1000 kHz at room temperature. Equation (1) was employed to compute the dielectric constant.

(1) $$\begin{array}{c} \varepsilon^{\prime }=\frac{C_{aps}}{C_{0}}. \end{array}$$

## 3. Result and Discussion

### 3.1. Dielectric Measurements

Each material has some capacity for storing electricity, which is determined by its dielectric constant. The capacitance is determined by the relative permittivity, which is known as the dielectric constant.

(2) $$\begin{array}{c} C=\frac{\varepsilon_{0}\;k\;A}{D}, \end{array}$$

where *C* is the capacitance of the parallel plate capacitor in Farad, *κ* is the dielectric constant, *ε*_0_ is the permittivity of the free space, *A* is the area of parallel conducting plates, and *D* is the separation between parallel conducting plates. It is thus feasible to infer a change in the dielectric constant of a material through a change in its capacitance which change through the magnitude of charge stored (*Q* = CV). The dielectric constant can be used to calculate the ability of the material to store a specific quantity of electricity or energy. Oils with higher dielectric constant and viscosity level have better biodegradability. Moreover, oils with higher dielectric constant are considered healthier because their biodegradability is linked to human health, but it is important to clarify those oils with high dielectric constant not necessarily desirable for some application need high dielectric constant at high frequencies. Generally, vegetable oils with high dielectric constants break down more easily when subjected to high electric field frequencies.

Ionic transport is the major method of DC conduction in olive oil. The impedance is high because there are few ions mostly from fatty acids, making olive oil a strong dielectric material. Clear space pathways are required for ionic transport. Triglycerides are composed of three long carbon chains that can be incorporated into other compounds. This makes ion transfer more challenging because the chains limit the ionic channels [21].

**Figure 3:**
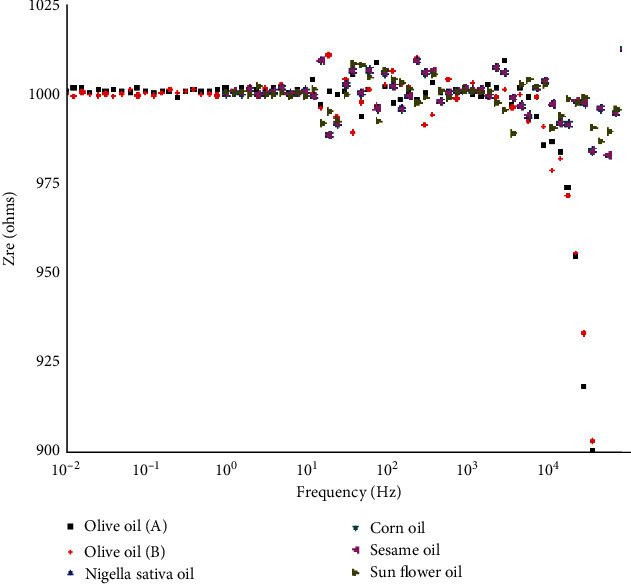
Frequency dependence of the real part of the impedance for all samples.

Figure 3 shows the dependence of the real part of the impedance (ZRE) on frequency for the various oils. In the low frequency range, *Z*_*RE*_ (i.e., resistance) is frequency independent. However, ZRE decreases as frequency increases in the high-frequency range (10^4^–10^5^ Hz), implying that AC conductivity will also increase. The higher-frequency domain merging of the real part of the impedance suggests a possible release of space charge, resulting in the reduction of material barrier characteristics [22].

**Figure 4:**
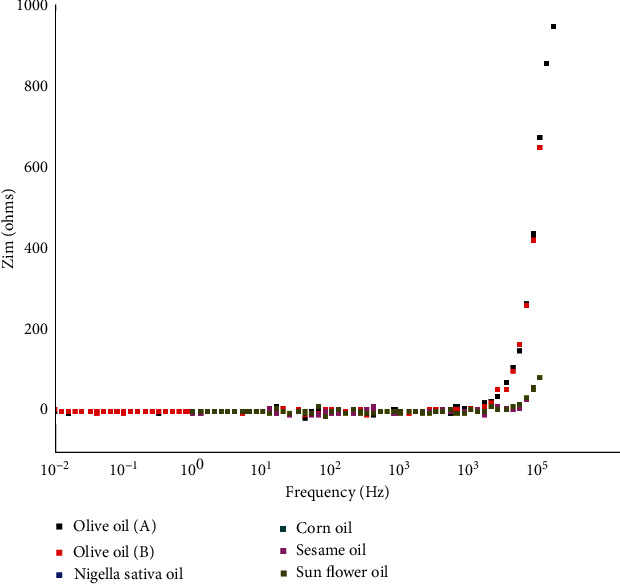
Frequency dependence of the imaginary part of the impedance for all samples.

Figure 4 shows the variation in the imaginary part of the impedance (*Z*_*Im*_) with frequency for the different oils. The magnitude of *Z*_*Im*_ is very low for low frequencies for all types of oils and then increases as the frequency increases, especially in the range of 3 × 10^4^–1 × 10^5^ Hz.

**Figure 5:**
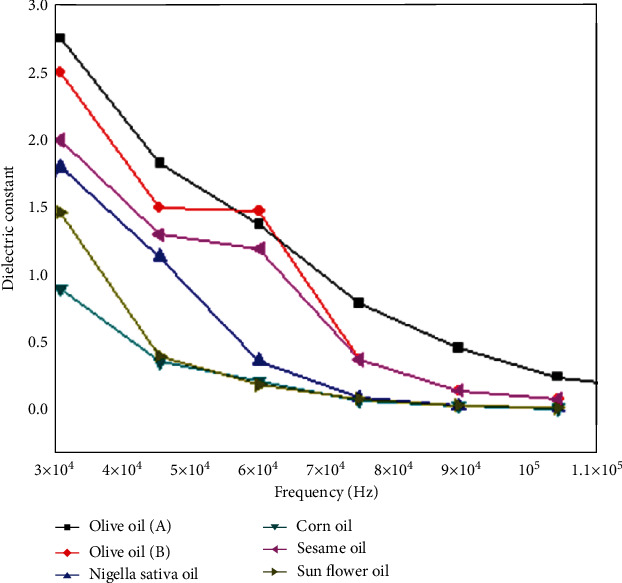
Frequency dependence of the dielectric constant for all samples.

Figure 5 illustrates the decrease in the dielectric constant for all the tested oils as a function of the frequency of the applied electric field. Because there is little time for the molecule to rotate at higher frequencies, the orientation polarization decreases with a tendency to disappear, and only the dipole polarization term contributes to the dielectric constant at these frequencies, which is expected for all oils [23]. However, considerable modifications were observed at low frequencies. For olive oil A, olive oil B, sesame oil, Nigella sativa, sunflower oil, and corn oil, the maximum values measured for the dielectric constant at lower frequencies were approximately 2.75, 2.5, 2.0, 1.75, 1.5, and 0.9, respectively.

### 3.2. Functional Group Analysis of Oil

Fourier transform infrared spectroscopy (FTIR) was used to analyze the functional groups present in the oils. Figure 2 shows the FTIR spectra of olive cooking oil A, olive cooking oil B, Nigella sativa, sesame oil, corn oil, and sunflower oil.

**Figure 6:**
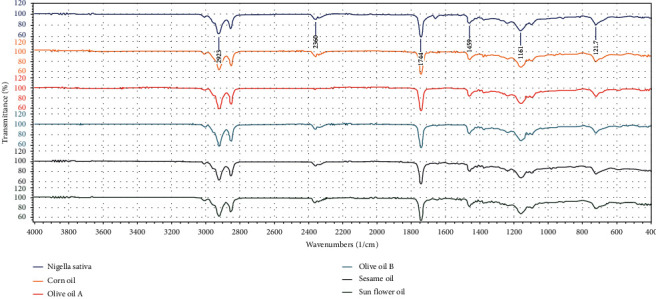
FTIR spectra of olive cooking oil A, olive cooking oil B, Nigella sativa, sesame oil, corn oil, and sunflower oil.

The FTIR profiles of olive cooking oil A, olive cooking oil B, Nigella sativa, sesame oil, corn oil, and sunflower oil were similar with multiple significant transmission bands. The FTIR spectrum of the oils revealed transmission bands at roughly 2923 and 2850 cm^−1^, indicating C-H stretching, as shown in Figure 6. This demonstrates that aliphatic hydrogen was present in all oil samples. There is a transmission band at approximately 1744 cm^−1^, indicating C=O stretching. This shows the presence of a carbonyl group in all oils, except for olive oil, which has a very weak transmission band with the same wavenumber. The methylene rocking vibration, in which all methylene groups rock in phase, is visible as a faint absorption band around 721.7 cm^−1^[24]. From 1800 to 990 cm^−1^ is the shoulder of carbonyl compounds, and band around 721.7 cm^−1^ may be due to cis/trans isomer form of fatty acids.

### 3.3. Dielectric Numerical Simulation

**Figure 7:**
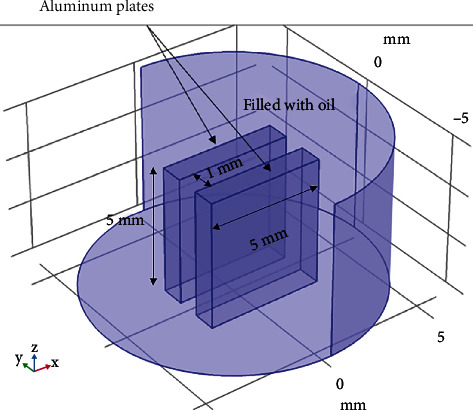
Three-dimensional geometry of the interelectrode capacitance filled with oil.

The proposed method was evaluated using the finite element method (FEM). FEM simulations of various olive oils were performed to calculate the interelectrode capacitance. The model was adequately simplified to facilitate the simulation process. COMSOL Multiphysics simulation software was used to perform the simulation process, as shown in Figure 7.

The dimension of the simulated capacitor is the same as in experimental (5 × 5 mm and a fluid gap of 1 mm in parallel mode). The electric field and device capacitance were solved under electrostatic conditions in this simulation, which provided a simple capacitor model [25]. The FEM can determine the electric potential distribution *V*(*x*, *y*, *z*) by applying an excitation voltage to one electrode and connecting the other electrodes to the ground. Equation (3) is used to calculate the interelectrode capacitance.

(3) $$\begin{array}{c} C=\frac{Q}{V\left(x,y,z\right)}, \end{array}$$

where *Q* is the charge. The dielectric constant can be obtained using the following electrical model

(4) $$\begin{array}{c} \varepsilon \left(\omega \right)=\frac{1}{\omega C_{0}Z^{\prime \prime }\left(\omega \right)}, \end{array}$$

where *C*_0_ = *ε*_0_*A*/*h* is the capacitance of the vacuum cell; *ε*_0_ = 8854 Pf/m is the permittivity of vacuum, *A* is the electrode surface area, *h* is the distance between the two electrodes, and *Z*′′(*ω*) is the imaginary part of the impedance.

The behavior of the electric field intensity (*E*) and electric flux density (alternatively, electric displacement, *D*) is considered in electrostatic problems. These quantities must comply with the following two requirements. The differential form of the Gauss's law states that the flux out of any closed volume is equal to the charge contained within the volume. Maxwell's equations are used to compute the potential (electric field). Equations (5) and (6) were used in the electrostatic model.

(5) $$\begin{array}{c} \nabla \cdot D=\rho_{v}, \end{array}$$

(6) $$\begin{array}{c} E=-\nabla v. \end{array}$$

**Figure 8:**
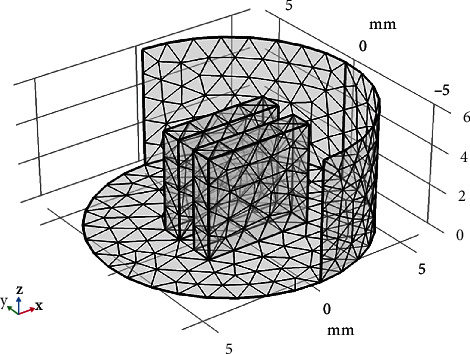
Free tetrahedral meshing geometry.

The next four steps in COMSOL Multiphysics for resolution can be characterized as follows:

- The first step is to set the three-dimensional (3D) geometry of the aluminum electrodes (with a diameter of 5 × 5 mm and a fluid gap of 1 mm in parallel mode) and several types of oil used as insulators (software).
- The second step is to define the meshing parameters, which are defined by default through the free tetrahedral meshing geometry, as shown in Figure 8.  Table 2 lists the meshing parameters

- The third step is to define the electrical properties of the materials employed. This entails the determination of the relative permittivity and conductivity of each component of the insulator. In addition, boundary conditions are defined, which are translated into the potential applied to each electrode (Dirichlet conditions)
- The fourth step is devoted to solving the differential form of Gauss's law can alternatively be written as [∇·(*ε*_0_∑_*r*_*E*) = *ρ*_*v*_], where ∇·*E* is the divergence of the electric field, *ε*_0_ is the vacuum permittivity, and *ρ* is the volume charge density (charge per unit volume)
- The problem is solved by applying the numerical method and the construction of the system of all previous equations, and this is done by introducing the factors of each part of the equation solved in stationary as solver configuration
- The final stage is to solve the problem and display the simulation findings as a potential and electric field distribution and to compare the computed dielectric constant to the corresponding experimental values

**Figure 9:**
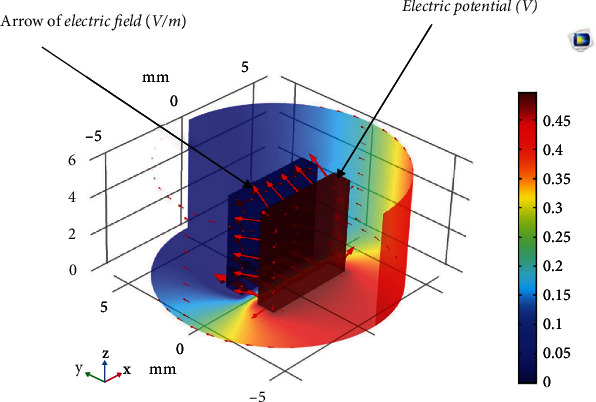
Variation of the potential and electric field along the distance inside the olive oil A sample (as an example).

Figure 9 shows the potential and electrical potential distributions for the olive oil sample, for example, the potential changes with the distance from the ground at the left electrode, where the potential is the greatest near the high-voltage electrode and diminishes as one approaches the ground electrode. The voltage calculated in this model is required for subsequent calculations of the imaginary impedance and oil dielectric constant.

**Figure 10:**
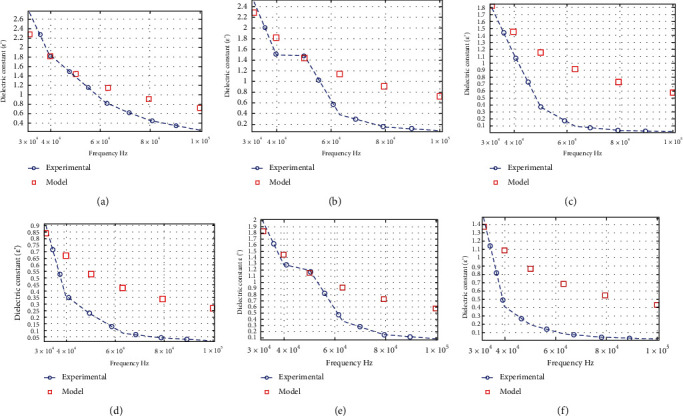
Comparison between the experimental and model results for frequency versus of dielectric constant of (a) olive oil A, (b) olive oil B, (c) Nigella sativa, (d) corn oil, (e) sesame oil, and (f) sunflower oil samples.

The simulation model must be validated to ensure confidence in the model results. The simulation results were compared with the experimental results to determine the validity of the dielectric constant model.  Figure 10 compares the experimental and simulation results for the frequency range of 10^4^–10^5^ Hz versus the dielectric constant of olive oil A, olive oil B, Nigella sativa, corn oil, sesame oil, and sunflower oil samples for the frequency range of 10^4^–10^5^ Hz. Although there is a mismatch between the model and practical applications, there is an acceptable convergence between them due to the difficulties in accurately estimating the physical and electrical properties of all oils.

**Figure 11:**
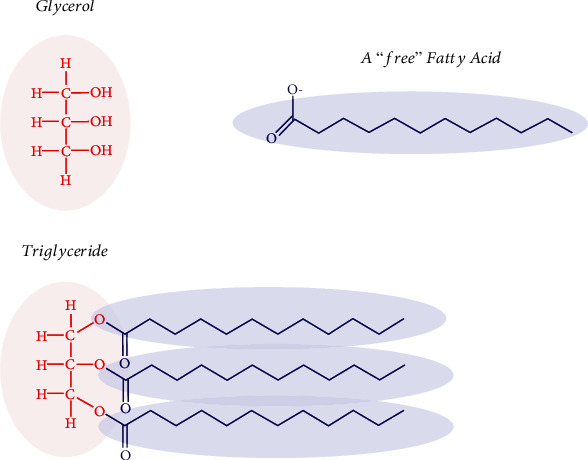
Olive oil triacylglycerol chemical formula [26].

Olive oils have greater dielectric constant values than other vegetable oils when the dielectric constant values of olive oils are compared to those of other vegetable oils. This may be due to the presence of long triglycerides in olive oils as shown in Figure 11, which are more polar than other vegetable oils. These compounds are naturally formed by esterification of three fatty acid molecules with a glycerol molecule.

The values for all oils decreased in both experimental and numerical results as the frequency increased. This shift is consistent with the Debye equations for the frequency dependence of the dielectric constant, where a significant decrease occurs with frequency. This declining trend is also consistent with previous findings [27], according to which the dielectric constant of selectively hydrogenated vegetable shortening showed a general plateau in the low-frequency range and then a reduction as the frequency increased. Research on the dielectric characteristics of oils in the low-frequency range will help create a simple and inexpensive method for determining oil quality. This change is only observed in vegetable oils (also known as dipole polarization) because of the orientation polarization of the dielectric medium.

## 4. Conclusions

Frequency dependency and specific values of the dielectric constant for each oil and fatty acid were found for a variety of edible oils. All oils investigated had a higher dielectric constant value at lower frequencies, which subsequently dropped dramatically at higher frequencies. At low frequencies, the measured maximum dielectric constants yielded approximate values of 2.75, 2.5, 2.0, 1.75, 1.5, and 0.9 for olive oil A, olive oil B, sesame oil, Nigella sativa, sunflower oil, and corn oil, respectively. The dielectric constant of the oils was computed using the COMSOL Multiphysics program, which was in good agreement with the experimental data in most cases. The functional groups found in the oils were analyzed using FTIR, which revealed that the spectral differences were modest. The findings of this study could be beneficial for identifying oils, evaluating their quality, and monitoring their quality during processing and storage.

## Figures and Tables

**Table 1:** Physicochemical properties of oils at room temperature 35 *σC*.

Properties	Olive oil A	Olive oil B	Sesame oil	Nigella sativa	Sunflower oil	Corn oil
Density (g/mL)	0.95	0.94	0.918	0.869	0.915	0.92
Viscosity ((mPa·s))	92	91	36	40	47	67
Boiling point (*σc*)	305	300	216	131	142	140
Saponification value (mg)	188	186	188	203	182	153

**Table 2:** Meshing parameters.

Description	Value
Minimum element quality	0.096
Average element quality	0.705
Tetrahedron	5742
Triangle	1080
Edge element	156
Vertex element	24
Maximum element size	1.2
Minimum element size	0.216
Curvature factor	0.6
Resolution of narrow regions	0.5
Maximum element growth rate	1.5

## Data Availability

Data collected from the literature can be consulted in the relevant articles; the authors' data are available upon request to Dr. Massaud Mostafa at mmostafa@ju.edu.sa
